# Interpersonal Neural Synchrony During Father–Child Problem Solving: An fNIRS Hyperscanning Study

**DOI:** 10.1111/cdev.13510

**Published:** 2021-01-10

**Authors:** Trinh Nguyen, Hanna Schleihauf, Melanie Kungl, Ezgi Kayhan, Stefanie Hoehl, Pascal Vrtička

**Affiliations:** ^1^ University of Vienna; ^2^ German Primate Center ‐ Leibniz Institute for Primate Research; ^3^ Georg‐August‐University Goettingen; ^4^ University of California, Berkeley; ^5^ Friedrich‐Alexander‐University Erlangen‐Nuremberg; ^6^ University of Potsdam; ^7^ Max Planck Institute for Human Cognitive and Brain Sciences; ^8^ University of Essex

## Abstract

Interpersonal neural synchrony (INS) has been previously evidenced in mother–child interactions, yet findings concerning father–child interaction are wanting. The current experiment examined whether fathers and their 5‐ to 6‐year‐old children (*N* = 66) synchronize their brain activity during a naturalistic interaction, and addressed paternal and child factors related to INS. Compared to individual problem solving and rest, father–child dyads showed increased INS in bilateral dorsolateral prefrontal cortex and left temporo‐parietal junction during cooperative problem solving. Furthermore, the father’s attitude toward his role as a parent was positively related to INS during the cooperation condition. These results highlight the implication of the father’s attitude to parenting in INS processes for the first time.

During the last decades, the paternal role has evolved substantially alongside major societal changes. It is widely agreed upon and supported by scientific studies today that children benefit from their fathers in numerous ways (Lamb, 2010). For example, fathers’ presence is associated with better cognitive development and greater perceived competence (Dubowitz et al., 2001). These new insights have been accompanied by the increasing recognition of fathers as caregivers and attachment figures by attachment theory where an important paradigm shift has occurred during the last two decades (Ahnert & Schoppe‐Sullivan, 2020), entailing a surge in research exploring the father–child dyad including father–child interactions (Bakermans‐Kranenburg, Lotz, Dijk, & van IJzendoorn, 2019). A recent study provided the first evidence for an association between fathers’ interactive behavior and variation in infant brain anatomy (Sethna et al., 2019), and other research linked experiencing fatherhood with neural changes in the father in brain areas associated with mentalizing and emotion processing (i.e., superior temporal sulcus or inferior frontal gyrus; see Bakermans‐Kranenburg et al., 2019). At the same time, and despite increasing knowledge on the relation between father–child interaction and children’s outcomes, little is known about the behavioral dynamics and brain mechanisms that possibly underlie this association. With the advancements of hyperscanning in parent–child interactions, interpersonal neural synchronization (INS)—suggested to support behavioral coordination and communication—has so far mostly been evidenced in mother–child interactions (Nguyen, Bánki, Markova, & Hoehl, 2020). Here, we present a functional near‐infrared spectroscopy (fNIRS) hyperscanning study investigating interpersonal neurobehavioral synchrony in father–preschool child dyads specifically.

During early social exchanges, behavioral coordination—such as synchrony—has been suggested to be essential to a child’s social, cognitive, and affective development (Feldman, 2012). More recently, this account was extended to also include synchrony on the physiological (e.g., heart rate, cortisol secretion) and neural (e.g., brain activity) levels (Feldman, 2017; Leclère et al., 2014). Focusing specifically on neural synchrony, the rhythmicity in behavioral coordination has been proposed to be reflected in the synchronization of neural oscillations (Hasson, Ghazanfar, Galantucci, Garrod, & Keysers, 2012; Markova, Nguyen, & Hoehl, 2019). As the behavioral communicative rhythms are transmitted through the environment (e.g., as speech sounds), the sensory system of one person is coupled to the motor system of another person. In both individuals of an interacting dyad, entrainment of internal neuronal oscillations to external rhythms has been shown to enable optimal processing of rhythmic stimuli, because sensory input is then sampled during phases of high neuronal co‐excitability (Calderone, Lakatos, Butler, & Castellanos, 2014). Accordingly, INS has been proposed as an essential mechanism to facilitate the transmission of information through verbal and non‐verbal communication between a dyad (or within groups; Dumas, Lachat, Martinerie, Nadel, & George, 2011; Hasson et al., 2012). A growing body of research indeed provides evidence for higher levels of INS during cooperative interactions to be associated with higher task performance in terms of joint goal achievement in adults as well as adult–child dyads (see Hoehl, Fairhurst, & Schirmer, 2020). These findings underscore facilitated coordination and communication in relation to increased INS for parent–child dyads. The above said, data on the neural aspect of bio‐behavioral synchrony during social interaction remain scarce. This generally applies for parent–child dyads, but particularly so for father–child dyads.

Existing hyperscanning studies using fNIRS have mostly investigated INS in mother–child dyads (Nguyen, Bánki, et al., 2020). fNIRS allows to measure brain activity, indicated by oxygenation changes in hemoglobin, in naturalistic interactions. It is less susceptible to motion artifacts while compromising on cortical depth and temporal resolution in comparison to fMRI and EEG/MEG, respectively (refer to Lloyd‐Fox, Blasi, & Elwell, 2010 for more information). In a growing body of research, INS in caregiver–child dyads—involving children of different ages from infancy to school age—has been observed during naturalistic problem solving (Nguyen et al., 2020a; Quiñones‐Camacho et al., 2019), non‐verbal dyadic interaction (Piazza, Hasenfratz, Hasson, & Lew‐Williams, 2020), free verbal conversation (Nguyen et al., 2020b), as well as standardized cooperative button‐press tasks (e.g., Reindl, Gerloff, Scharke, & Konrad, 2018). These studies provide first evidence for INS during caregiver–child interaction for different child age groups from infancy to adolescence and across a range of different interaction contexts. Common to all published studies with children is that INS varied as a function of mutual engagement across different behavioral modalities (i.e., gaze, speech, motor) in the given task or interaction. In contrast, when assessing behavioral coordination, the relevant modalities change in their relevance as children develop. For example, conversational coordination only emerges when children can verbalize themselves (Feldman, 2012). We thus propose INS as a useful biomarker for mutual engagement in parent–child interaction. Studying INS in father–child dyads can help us better understand the unique features and commonalities in mother–child and father–child interactions and may unveil essential mechanisms to interpersonal behavioral coordination throughout early childhood.

Studies on mother–child cooperation and communication consistently report INS in the temporoparietal junction (TPJ) and lateral prefrontal cortex (PFC), even though different estimation methods for INS were used (see Nguyen, Bánki, et al., 2020). INS in the TPJ has been implicated in interpersonal behavioral coordination, which is essential to parent–child interactions (Hoehl et al., 2020; Leclère et al., 2014). Interpersonal alignment of rhythms during coordination depends on various influencing factors, notably their socio‐emotional value, computed in temporoparietal regions including the TPJ (Koster‐Hale & Saxe, 2013). Besides the tracking of socio‐emotional value of incoming visual information and engagement in mental state representation associated with TPJ functions, cooperation also involves top‐down cognitive control over emotional processes in social contexts. This function is associated with the engagement of a cognitive control system mainly localized in lateral prefrontal areas (i.e., dorsolateral prefrontal cortex [dlPFC]; Balconi & Pagani, 2015; Long, Verbeke, Ein‐Dor, & Vrticka, 2020). Thus, INS in the dlPFC is suggested to represent a potential biomarker for mutual attention and shared intentionality during social interactions, as well as to reflect emotion regulation abilities (Gvirts & Perlmutter, 2020; Reindl et al., 2018). Overall, the emerging findings suggest a prominent role of the TPJ and dlPFC in INS during caregiver–child interactions.

Although fathers are increasingly recognized as caregivers and attachment figures to their children, parent–child hyperscanning studies focusing on or including father–child interactions are scarce. The available behavioral data suggest that fathers’ and mothers’ interactional behavior toward their children should in principle not substantially differ. For example, father– and mother–child dyads appear to show similar levels of interpersonal behavioral synchrony during their interactions (de Mendonça, Bussab, & Kärtner, 2019; Feldman, 2003), and father– and mother–child interaction quality seems to be comparable more generally (e.g., Piskernik & Ruiz, 2020). These results suggest that father–child dyads might show comparable associations of INS to state‐like factors, such as behavioral reciprocity, as mother–child dyads.

Besides state‐like factors relating to behavioral synchrony of the dyad, other state‐like factors concerning parent characteristics have been probed. Two studies revealed maternal stress to attenuate INS in mother–child dyads independent of the interactive context (Azhari et al., 2019; Nguyen et al., 2020a). Conversely, maternal sensitivity as another predictor of parent–child relationship quality (Leclère et al., 2014) could so far not be linked to INS—although such lack of association may be due to too little variance in the assessed high socioeconomic status sample (Nguyen et al., 2020a). Similar qualities of paternal behavior also seem to affect children’s development (Brown, Mangelsdorf, & Neff, 2012). More precisely, fathers’ involvement in child care is related to fathers’ sensitivity in caregiving and subsequent positive outcomes in child development (Cowan, Cowan, Pruett, & Pruett, 2019; Flouri, Midouhas, & Narayanan, 2016). More generally, the trait‐like factor of father involvement is not only suggested to positively affect fathers’ caregiving behavior but also to change fathers’ physiology (Bakermans‐Kranenburg et al., 2019). In contrast, perceptions of distress related to parenting may shape fathers’ behavior in ways that have a negative impact on children and may even put them at risk for psychopathology (e.g., Barker, Iles, & Ramchandani, 2017). Taken together, these findings emphasize the need to take state‐like factors like paternal sensitivity and distress, as well as trait‐like factors like father involvement into account when studying father–child interactions using hyperscanning in the context of bio‐behavioral synchrony.

Additionally, results from other fNIRS hyperscanning studies corroborate findings from behavioral research highlighting the increasingly active role of the child during interactions at preschool age (Harrist & Waugh, 2002; Nguyen et al., 2020a). While Nguyen et al. (2020a) observed that child agency, a state‐like factor, could facilitate INS within mother–child dyads, Quiñones‐Camacho et al. (2019) emphasize the role of child irritability during recovery phases of stressful interactions as potentially inhibiting task‐related INS. Moreover, the biological sex of the child seems to be related to the interaction qualities of father–child dyads (de Mendonça et al., 2019). Crucially, however, the evidence here is somewhat inconsistent. For example, while father–daughter dyads were observed to be more attuned with fathers showing more sensitive, structuring, and non‐intrusive behavior toward their daughters (e.g., de Mendonça et al., 2019), other observations indicated that fathers are more involved with and responsive to their sons (Feldman, 2003). Also, earlier studies suggest that fathers are more likely to support active play in boys than in girls (Tauber, 1979) and father–son interactions are marked by higher child agency without getting into conflict or competition (Buss, 1981). Combined, there seem to be some inconsistencies in behavioral findings indicating that father–child dyads may be characterized by similar—but not necessarily equal—patterns in INS and interaction quality as compared to mother–child dyads. At the same time, available findings indicate that father–child dyads may likely show sex differences in their interaction qualities. Henceforth, it appears that child biological sex needs to be considered in the investigation of father–child INS.

In the present study, we specifically focused on the question of whether father–child dyads show INS during cooperative problem solving measured with fNIRS hyperscanning. The task was contrasted with an individual problem‐solving control condition as well as rest phases. We were able to build on the preexisting literature with mother–child dyads using the tangram puzzle task (Nguyen et al., 2020a) to test whether father–child dyads would show similar patterns of INS during problem solving, including the state‐ and trait‐like factors already assessed within this context. The fact that we exclusively focused on fathers as the caregiver during these interactions allowed us to deepen our understanding of the neurobiological underpinnings of parent–child interactions in general, and provided the opportunity to investigate potential factors specific to father–child dyads either facilitating or attenuating neurobehavioral synchronization. Moreover, understanding how similar patterns in both behavior and brain‐activity may conform but also differ between mother–child and father–child dyads may hopefully shed light on the unique contribution of fathers for child development.

We investigated the following main research questions:

1. Do fathers and children show increased INS during the cooperative problem‐solving task in comparison to individual and resting phases? Given above‐cited evidence that increased INS in the TPJ and dlPFC has been found in several mother–child studies with a range of child ages (e.g., Nguyen et al., 2020a; Reindl et al., 2018), we expected INS in the same brain areas to be the highest during the cooperation condition (in comparison to all other conditions).
2. Is INS during cooperation related to dyadic behavioral variables, that is, task performance and behavioral reciprocity? The link between INS and task performance has not only been evidenced in mother–child studies but also adult dyads in various forms of cooperative tasks ranging from button‐press to problem solving (Nguyen, Bánki, et al., 2020). We therefore also predicted a positive link between task performance and INS in all regions of interest during cooperation in father–child dyads. Furthermore, evidence from recent developmental research suggests higher levels of behavioral reciprocity to be associated with higher levels of INS in the TPJ and dlPFC during cooperative mother–child problem solving (Nguyen et al., 2020a; Quiñones‐Camacho et al., 2019). We therefore hypothesized father–child dyads to show the same association in the same brain areas.
3. Is there an association between INS during cooperation and child agency? The role of child agency was previously evidenced by an association with higher INS in the TPJ and dlPFC during mother–child cooperation (Nguyen et al., 2020a). We therefore assumed that this association would show in father–child cooperation as well.
4. What are the parental state‐like behavioral and self‐report markers of INS in father–child cooperation? We assumed paternal sensitivity to be positively related to INS during cooperation. Although maternal sensitivity was not significantly related to INS during cooperation in a previous study (Nguyen et al., 2020a), we expected fathers’ sensitive caregiving to provide unique individual differences in association with INS in both the TPJ and dlPFC during cooperative problem solving. Conversely, according to previous literature indicating that maternal stress may be associated with decreased INS in the TPJ and dlPFC during cooperation (Azhari et al., 2019; Nguyen et al., 2020a), we predicted a similar pattern in the present father–child dyads.
5. Are there any associations between INS during cooperation and more trait‐like characteristics associated with fatherhood? As fathers’ sensitivity and involvement in child care were found to be related to positive outcomes in child development more generally (Cowan et al., 2019; Flouri et al., 2016), we also expected a positive relation between fathers’ appreciation of their role as a parent (measured with the Role of the Father Questionnaire [ROFQ]—see below) and INS in all regions of interest during cooperation.

The present study and the above‐mentioned hypotheses were preregistered on aspredicted.com (https://aspredicted.org/u84z6.pdf). We furthermore exploratively evaluated the associations between father–child INS and (a) dyadic and individual behavioral patterns as the well as (b) biological child sex. These additional exploratory analyses were not preregistered.

## Method

### Sample

Sixty‐six fathers (*M* = 39.2 years, *SD* = 5.17 years) and their preschool children (*M* = 5.32 years, *SD* = 0.31 years; 31 girls) participated in the present study. Out of the initially recruited 68 dyads, 2 were excluded due to non‐compliance with the given instructions (*n* = 1) and refusal of the child to wear the fNIRS cap (*n* = 1). Optimal sample size calculations using G*Power for a medium effect size repeated measures design (groups = 3, *f* = .25, α = .05, 1‐beta = .90) resulted in *N* = 36. The fNIRS assessment was combined with a subsequent fMRI experiment as well as attachment interviews (not reported here), with a predetermined sample size of *N* = 50 mother–child dyads with usable data available from all measurements. This cut‐off was reached after performing *N* = 68 fNIRS scans. Data collection took place from May 2018 to July 2019. Fathers were recruited from a database of volunteers based in and around a mid‐sized city in eastern Germany. All dyads were of White European origin and came from middle to upper‐class families based on parental education and family monthly income. Fathers had on average 6.84 years of higher education and 66.7% of families had a monthly income higher than 3,000€. Fathers were remunerated and children received a small gift for participating. Fathers provided written consent for themselves and their children and all procedures were approved by the local ethics committee.

### Procedure

**Figure 1:**
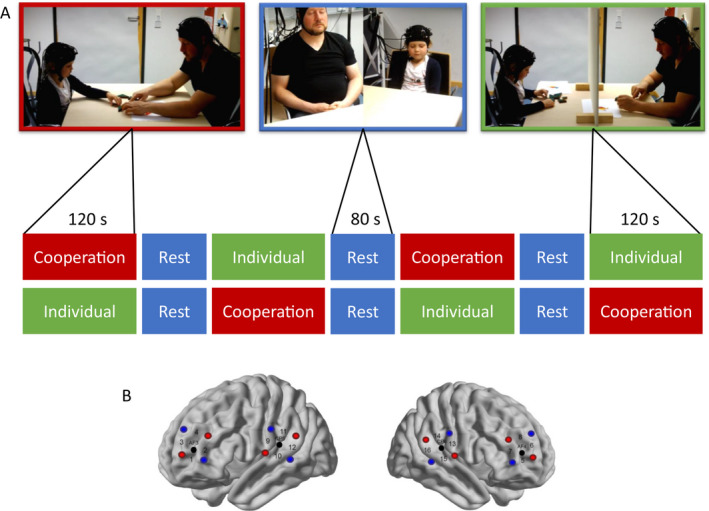
Experimental paradigm. (A) Illustration of the sequence of the experimental procedure showing an exemplary father–child dyad solving the tangram in cooperation (red) and individually (green). The rest phases are shown in blue. The order of conditions was counterbalanced and thus resulted in two possible sequences. (B) Illustration of the optode configuration at bilateral dorsolateral prefrontal cortex and temporo‐parietal junction anatomical locations (sources = red, detectors = blue, channel = numbers, EEG position = black). The left hemisphere is visible on the left, while the right hemisphere is depicted on the right.

Fathers and their children were welcomed to the lab and led to a testing room. After giving written informed consent the dyad was seated face‐to‐face, separated by a table, and was guided through a cooperative problem‐solving condition (120 s), an individual problem‐solving condition (120 s), and 80 s of rest with eyes closed in between each condition (see Figure 1A). Cooperation and individual problem solving were repeated twice and the order was counterbalanced. In the problem‐solving conditions, father and child were instructed to either cooperatively or individually arrange tangram puzzles and recreate templates of abstract forms, objects, and animals (see Nguyen et al., 2020a for more information). During the individual condition, an opaque screen was put in between the dyad to help the caregiver and the child to focus on their own puzzle. In the rest phases, father and child were instructed to close their eyes, relax, and refrain from talking to each other. Subsequently, the child had to solve a preschool form (this task will not be reported further here). The whole procedure was recorded on video from three different angles capturing frontal images from the father and the child and one of the whole dyad from the side.

### fNIRS Acquisition

We recorded oxy‐hemoglobin (HbO) and deoxy‐hemoglobin (HbR) concentration changes for each dyad using a NIRScout 8 × 16 (NIRx Medizintechnik GmbH: Berlin, Germany) Optical Topography system. Eight light sources and eight detectors were grouped into four 2 × 2 probe sets and were attached to an EEG cap with 10–20 configuration. The probes were placed according to standard electrode locations. To assess brain activity in the left and right dlPFC electrode locations for AF3 and AF4 were used for guidance, whereas the probes over the left and right temporo‐parietal junction (TPJ) were placed according to CP5 and CP6. The regions of interest (ROI) were based on previous studies investigating cooperative parent–child interactions (Nguyen et al., 2020a; Reindl et al., 2018). The four‐probe sets resulted in 16 measurement channels with equal distances of 3 cm between the optodes. The absorption of near‐infrared light was measured at the wavelengths of 760 and 850 nm and the sampling frequency was 7.81 Hz.

### fNIRS Processing

Before analyzing the fNIRS measurements, raw data were visually inspected during an initial quality check procedure. This resulted in 2.16% of the channels from the whole sample to be removed from further analyses. After this initial step, remaining data were pre‐processed using MATLAB‐based functions derived from SPM for fNIRS (Tak, Uga, Flandin, Dan, & Penny, 2016). Raw optical density values were first motion‐corrected with MARA, a smoothing procedure based on local regression using weighted linear least squares and a 2nd‐degree polynomial model (Scholkmann, Spichtig, Muehlemann, & Wolf, 2010), and then filtered with a high‐pass parameter of 0.01 Hz (Reindl et al., 2018). Next, the filtered data were converted to HbO and HbR values based on modified Beer‐Lambert Law with age‐dependent differential path length factors. In the following statistical analyses, we focused on HbO values, which were reported to be more sensitive to changes in the regional cerebral blood flow (Hoshi, 2016). Statistical analyses for HbR are included in the Appendix S1.

### Wavelet Transform Coherence

INS was estimated using Wavelet Transform Coherence (WTC) based on the Morlet wavelet (Chang & Glover, 2010; Grinsted, Moore, & Jevrejeva, 2004 for more information). WTC estimates a coherence coefficient between two fNIRS time series based on frequency and time and thus results in a synchrony score comprising both in‐phase and phase‐lagged synchrony in a certain frequency band. WTC is the most commonly used estimation approach to INS (see Czeszumski et al., 2020) and includes both time‐related as well as frequency‐related properties of the two time‐series. The frequency band of interest for this study was determined to be 10–50 period seconds (approx. 0.08–0.1 Hz) based on visual inspection and a previous study using the same paradigm (see Figure A1 in Appendix S1; Nguyen et al., 2020a). WTC values were averaged across conditions and frequency bands to result in 16 (channels) × 4 (conditions) INS values. For all four conditions the same length of data, namely 240 s, went into the calculation. We further conducted a random pair analysis with 1,000 permutations to rule out effects due to spurious correlation ‐ please refer to the Appendix S1 for further details.

### Behavioral Task Performance and Interaction Quality

Individual and dyadic task performance were indexed by the numbers of templates solved during the individual and cooperation condition, respectively. Interaction quality, namely behavioral reciprocity, parental sensitivity, and child agency, during the cooperation condition was rated by three graduate students from the video recordings using a coding scheme adapted from a previous study investigating INS during mother–child problem solving (Nguyen et al., 2020a). Shortly summarized, ratings of child agency and behavioral reciprocity were derived using a customized coding scheme based on the Coding System for Mother–Child Interactions (Healey, Gopin, Grossman, Campbell, & Halperin, 2010), and paternal sensitivity was assessed with a German instrument labeled INTAKT (an agglomeration of the German word “Interaktion” meaning interaction, and “intakt” meaning intact—referring to an intact mother child relationship; Hirschmann, Kastner‐Koller, Deimann, Aigner, & Svecz, 2011). For more details on these coding scales (as well as interrater reliability from 25% of the video recordings calculated as intraclass correlations—ICC), please refer to Table 1. Each scale was rated on a 7‐point Likert‐type scale (1 = *no occurrence*, 7 = *continuous occurrence*) and averaged over the two cooperation conditions. Any discrepancy of more than one point on each scale between coders was reviewed and a consensus was obtained.

**Table 1:** Scale Names, Descriptions, and Interrater Reliability Are Depicted in the Table

Subscales	Description	Intraclass correlation
Cooperative task performance	Cooperative task performance is indicated by the number of templates solved during the cooperation condition	—
Individual task performance	Individual task performance is indicated by the number of templates the child solves by him or herself during the individual problem‐solving condition	—
Behavioral reciprocity	Contingent behavioral responses to the interaction partner and mutual engagement in the task. Higher ratings indicate turn‐taking and that the dyads were attentive to one another. They took interest and pleasure in the mutual task completion as well as in the interaction. Furthermore, dyads with high scores displayed reciprocal behaviors coupled with signs of shared affect, such as smiling or eye contact. Dyads scoring low on the scale are characterized as being impatient or having disregard for the partner’s actions, being passive, or completing the task in parallel without any shared experience	*r* = .74
Paternal sensitivity	The scale entails the father’s prompt, appropriate, and sensitive response to his child’s signals. Fathers who scored high on this scale were continuously oriented towards the child’s needs and wishes. They were loving and warm and gave appropriate and supportive feedback in a way that motivated the child. Low sensitivity was characterized as low emotional engagement with the child or in the interaction, and being insensitive to the child’s cognitive and emotional needs	*r* = .83
Child agency	This scale captures how the child approaches the task. High scores were assigned to a child that shows interest, vigor, enthusiasm, and eagerness to do the tasks. The child invested efforts in his or her activities, was confident and valued success. Moreover, high scores indicate that the child took on a leading role. Low scores imply a lack of confidence, interest or excitement, hesitant behavior, or restrained affect	*r* = .89

### Additional Trait‐Like Variables From Self‐Reports Related to Fatherhood

#### Role of the Father Questionnaire

Fathers’ attitudes toward fatherhood (i.e., the extent that fathers believe to play an important role for child development) were measured using a German translation of the ROFQ (Palkovitz, 1984) adapted to fathers of preschoolers. The ROFQ contains 15 items and fathers indicate their level of agreement or disagreement with each item on a 5‐point scale. Higher scores reflect attitudes that fathers are capable of caring for children and that they should be involved with and act sensitively toward their children. Internal consistency was adequate with Cronbach’s α = .78.

#### Parenting Stress Index

The current amount of fathers’ parenting stress was assessed with the short German version of the Parenting Stress Index (Tröster, 2011). This self‐report questionnaire evaluates the magnitude of parental stress within the child and the parenting domain. It consists of 48 items that form brief statements on stressors regarding the parent’s perception of the child and the parent’s functioning using a 5‐point Likert scale ranging from 1 (*not at all*) to 5 (*very strong*). Internal consistency was high with Cronbach’s α = .91.

### Statistical Analysis

Statistical analyses were run in RStudio (RStudio Team, 2020). Behavioral and questionnaire data analysis was conducted using linear regressions for behavioral scales, that is, task performance, parental sensitivity, child agency, engagement, as well as self‐report scales, that is, role of the father and parental stress. Behavioral reciprocity was analyzed using zero‐inflated Poisson regressions. We corrected *p*‐values with the false discovery rate (FDR; *q* < .05) for multiple comparisons. Descriptive statistics and results of linear regressions are reported in the Appendix S1.

For the INS analysis across our four ROIs and three conditions, a linear mixed‐effects model was calculated with the package lme4 (Bates, Mächler, Bolker, & Walker, 2015). As WTC values ranged from 0 to 1, they were Fisher’s *z* transformed. WTC values were entered as the response variable with condition (cooperation vs. individual vs. rest) and the interaction effect of ROI (four per dyad) as fixed factors and with random slopes for each condition in each dyad. For each a priori hypothesis, an individual statistical model was calculated. To test for the effects of individual and dyadic factors on INS beyond looking at the main pattern of INS (Model 1), we estimated the following statistical models using a subset of data containing only WTC values from the cooperation condition versus the individual condition (the latter serving as an active control condition) according to our pre‐registration. Model 2 (dyadic interaction variables) included the fixed and interaction effects of condition, cooperative task performance and behavioral reciprocity. Model 3 included the fixed and interaction effects of condition and child agency. Model 4 (state‐like paternal variables) comprised the fixed and interaction effects of condition, paternal sensitivity and parental stress. Model 5 (trait‐like paternal variables) included the fixed and interaction effects of condition and the Role of the Father. All model formulae and further details on model output are included in the Appendix S1.

Model fit was obtained comparing the full models that included all predictors with the respective null models that only included the random effect structure using a Likelihood ratio test (Dobson, 2002). To examine the significance of fixed effect factors, we calculated confidence intervals with the function confint to estimate robustness (i.e., confidence intervals excluding 0) and post‐hoc comparisons using the package emmeans to further analyze the relations according to our pre‐registered hypotheses. *p*‐values in post‐hoc contrasts were corrected using Tukey’s honest significant differences (Abdi & Williams, 2010).

## Results

### Interpersonal Neural Synchrony

**Figure 2:**
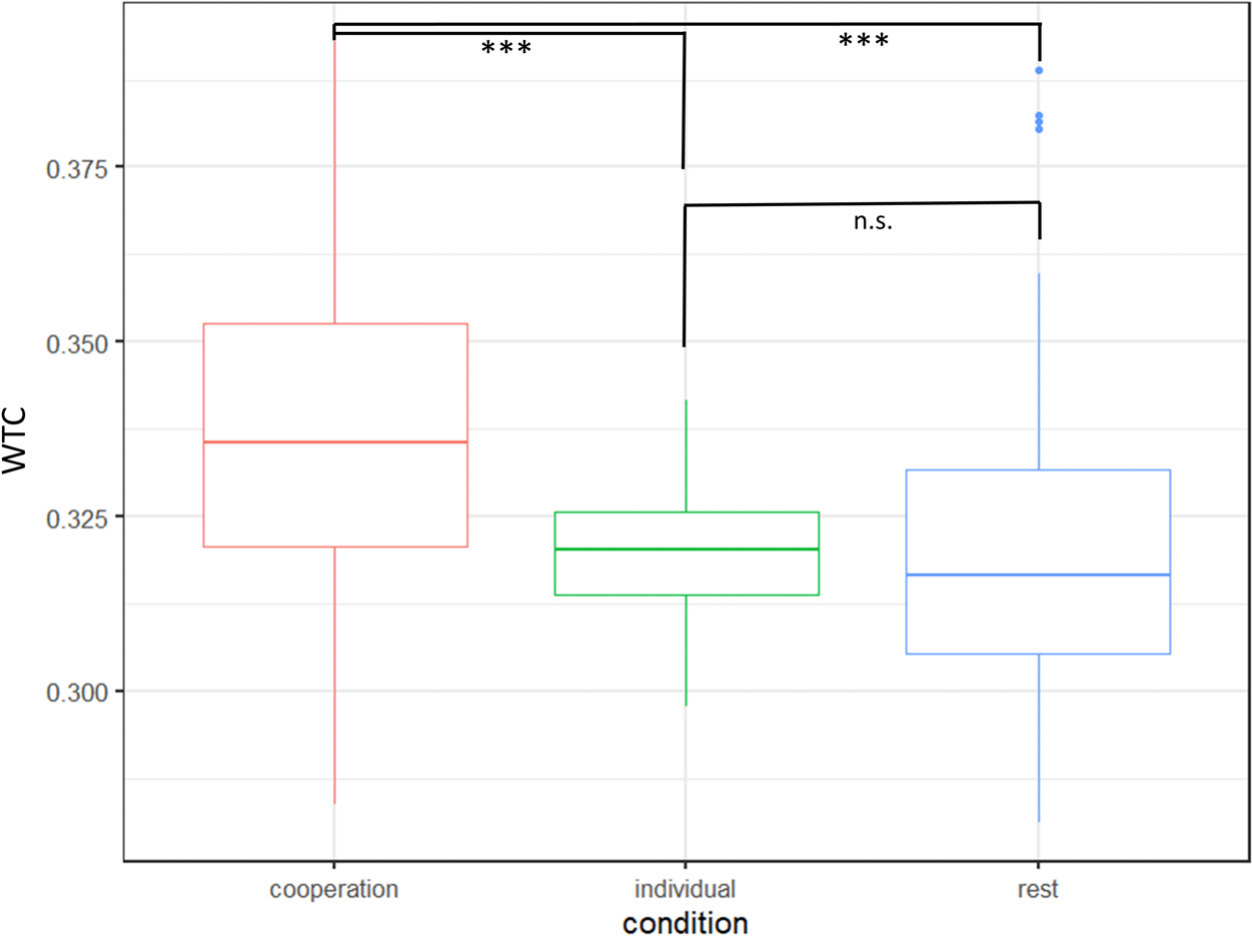
Illustration of interpersonal neural synchronization (INS; *y* axis) in terms of Wavelet Transform Coherence (WTC) during the cooperation condition (red) was significantly higher than in the individual (green) and rest (blue) conditions (*x* axis). ***p < .001.

First, we examined whether different father–child contexts of interaction and non‐interaction affected dyadic INS in dlPFC and TPJ (Model 1). Linear mixed‐effects analysis revealed a significant main effect of condition, χ^2^(3) = 22.34, *p* < .001 (see Figure 2). Overall, dyads showed higher levels of INS, in terms of increased WTC, during the cooperative problem‐solving task in comparison to individual problem solving as well as rest, *t* = 4.31–5.02, *p* < .001. We found no significant difference between INS during individual problem solving and rest, *p* = .984.

**Figure 3:**
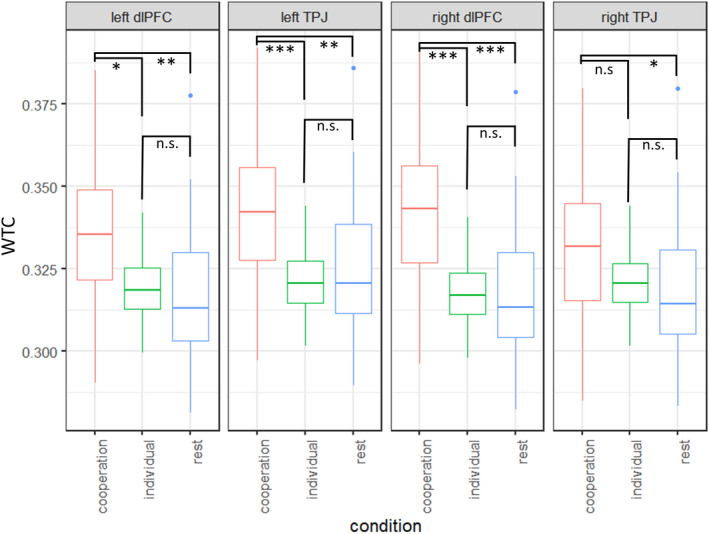
Illustration of interpersonal neural synchronization (INS; *y* axis) in the cooperation condition (red) as compared to the individual (green) or rest (blue) conditions (*x* axis) in bilateral dorsolateral prefrontal cortex (dlPFC) and bilateral left temporo‐parietal junction (TPJ; region of interest = facet columns). WTC = Wavelet Transform Coherence. *p < .05. **p < .01. ***p < .001.

Model 1 also resulted in a significant main effect of ROI, χ^2^(3) = 9.13, *p* = .027. However, the interaction between condition and ROI remained non‐significant, *p* = .121. Subsequent planned exploratory contrasts between conditions were calculated in each ROI and showed significant increases in INS during the cooperation as compared to the individual and resting conditions in the left and right dlPFC as well as left TPJ, *t* = 2.83–4.55, *p* < .002 (see Figure 3). Contrasts between conditions in the right TPJ were found to be significant for cooperation versus rest, *t* = 2.34, *p* = .049, but only marginal for the cooperation versus individual condition, *p* = .096. Overall, INS was increased in the cooperation condition in all ROI in comparison to an individual condition and rest phases. Subsequent analyses included the fixed effect of ROI to control for confounds.

### Dyadic Interaction Variables

Model 2 tested the effects of dyadic interaction variables, namely task performance and behavioral reciprocity, and whether they differed in their relation to INS in the cooperation versus the individual condition (the latter serving as an active control condition). Findings revealed non‐significant main effects and interactions for all predictors, *p* > .12, which means that in the current investigation in father–child dyads, cooperative INS was neither related to dyadic differences in behavioral reciprocity nor task performance.

### Child Agency

In a third linear mixed‐effects model (Model 3), we probed child agency ratings as fixed effects predictors. Results revealed that child agency was not related to INS in any condition as there were no significant main effects and interactions, *p* > .221.

### Paternal Variables

#### State‐Like Variables: Paternal Sensitivity and Parental Stress

In linear mixed‐effects Model 4, we tested the effects of state‐like paternal variables, that are, paternal sensitivity and parental stress in fathers. Model 4 revealed no significant main or interaction effects involving paternal sensitivity, *p* > .572, and analyses pertaining to parental stress only showed a marginal main effect, χ^2^(1) = 2.82, *p* = .092, estimate = −.004, *SE* = .003, 95% CI [−.009, .002]. Although there seemed to be previous indications for higher self‐reported parental stress to be related with lower levels of INS during mother–child interaction, this effect did not seem to be robust enough in the present sample of father–child dyads.

#### Trait‐Like Variables: Role of the Father

**Figure 4:**
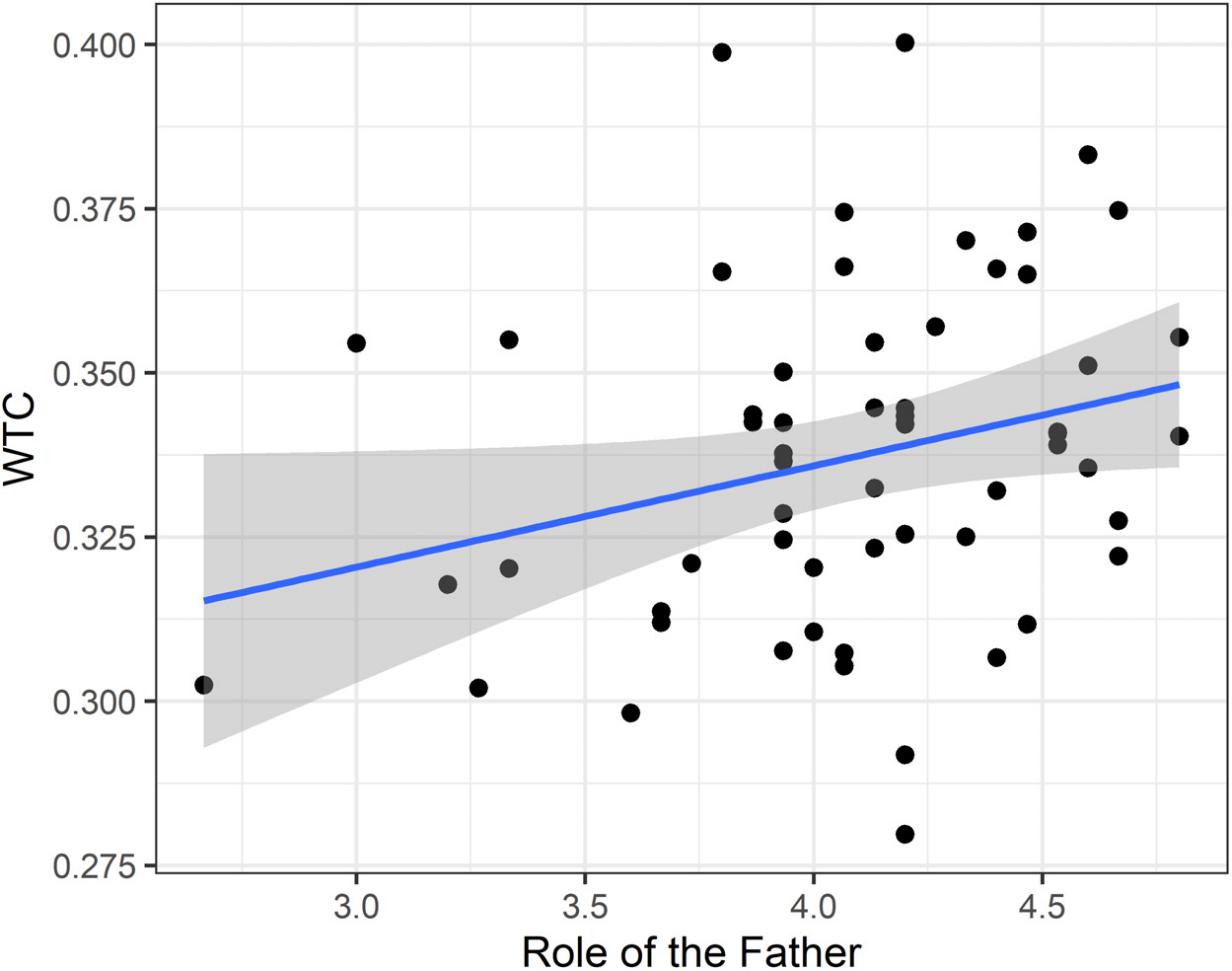
Illustration of the significant positive association between interpersonal neural synchronization (INS; *y* axis) in terms of Wavelet Transform Coherence (WTC) and Role of the Father Questionnaire (ROFQ) scores (*x* axis). The gray shaded area corresponds to 95% confidence intervals and each black dot corresponds to data from one dyad.

In Model 5 the effect of the trait‐like variable Role of the Father on INS was analyzed. Model 5 revealed a significant main effect of the Role of the Father, χ^2^(1) = 4.57, *p* = .033, as well as an interaction with condition that was approaching significance, χ^2^(1) = 3.59, *p* = .058 (see Figure 4). A subsequent post‐hoc trend analysis revealed the association to be positive and the most robust in the cooperation condition, estimate = .007, *SE* = .003, 95% CI [.002, .013], while positive but not robust in the individual condition, estimate = .002, *SE* = .003, 95% CI [−.003, .008]. This pattern indicated that when the father's attitude toward his role in child‐rearing was more involved, sensitive, and positive, INS in the cooperation condition was increased.

Results of the exploratively evaluated associations between dyadic and individual behavioral patterns as the well as the role of the biological sex of the child on father–child INS are reported in the Appendix S1.

## Discussion

In the present study, we investigated whether father–child dyads show interpersonal neural synchronization (INS) in temporo‐parietal and dorsolateral prefrontal areas in different interactive and non‐interactive contexts. Furthermore, we tested a number of dyadic, parental, and child state‐ and trait‐like characteristics hypothesized to associate with INS. Overall, father–child dyads showed increased INS in bilateral dlPFC as well as left TPJ during a cooperative problem‐solving task in comparison to individual problem solving as well as rest phases. This finding specifically in father–child dyads is consistent with previous literature assessing INS during mother–child cooperation at both preschool and school age (see Nguyen, Bánki, et al., 2020). In addition, we identified a significant association between Role of the Father Questionnaire (ROFQ) scores and INS during father–child interaction, being strongest during cooperation ‐ a pattern that again aligns with previous findings from mother–child interaction (Nguyen Kayhan, Matthes, Vrtička, et al., 2020). However, in contrast to extant studies (Nguyen et al., 2020a; Reindl et al., 2018), INS during cooperation in father–child dyads was unrelated to task performance, behavioral reciprocity or child agency. To conclude, our study is the first to underscore that differences in father–child neural synchrony could be related to differences in the trait‐like parenting attitude of fathers.

In line with previous fNIRS hyperscanning studies between adults (see Czeszumski et al., 2020 for a review) and caregiver–child dyads with mostly mothers participating (Nguyen, Bánki, et al., 2020), we find that fathers and preschool‐aged children show increased INS in the left TPJ as well as bilateral dorsolateral prefrontal areas (dlPFC) during cooperative problem solving. The TPJ is suggested to continuously track the socio‐emotional value of temporal regularities in sensory input, such as those induced through behavioral coordination necessary for cooperation (Hoehl et al., 2020). In addition, activation of the TPJ was found to be associated with social connectedness (Eddy, 2016). The dlPFC, on the other hand, has been implicated in cognitive top‐down control during cooperation to ensure that attention is directed toward task‐relevant information (Gvirts & Perlmutter, 2020). Temporal contingency in cognitive control likely reflects similar, mutually adapted cognitive demands and/or effort. Interestingly, a growing body of hyperscanning research shows that synchronised activity in the TPJ and dlPFC constitutes an important component of the neural mechanisms supporting social interaction (Redcay & Schilbach, 2019).

On a more general note, increased INS in TPJ and dlPFC during cooperation is suggested to be maintained to facilitate attunement and greater allocation of attention to the interaction to reap its potential benefits (Gvirts & Perlmutter, 2020). INS could not only facilitate mutual focus on the given task, but also increase salience of communication cues, thereby enhancing the predictability of social interactions. The more interactions are rhythmic and thus regular, the more they are predictable for both partners. With increased predictability through interpersonal synchrony it becomes easier for one individual to align their own rhythm to the rhythm of another person (Hoehl et al., 2020; Koban, Ramamoorthy, & Konvalinka, 2019). Accordingly, it was proposed that the rhythmic alignment of brain activity may reflect the regularity of interactive rhythms induced by social interactions (Markova et al., 2019). Increased INS in parent–child dyads during cooperation in the left TPJ and bilateral dlPFC may thus also relate to processes involved in processing and prediction of the interaction partner’s behavior (see also Perner, Aichhorn, Kronbichler, Staffen, & Ladurner, 2006), which has to be continuously monitored and adjusted to one’s own intentions and behavior.

Crucially, although the overall pattern of INS across bilateral dlPFC and TPJ was comparable in mother– and father–child pairs, the anatomical distribution of effects does not appear to be identical (Nguyen et al., 2020a). Father–child dyads only showed marginally significant increases in INS during cooperation in the right TPJ, which is generally linked to mentalizing and shared intention (Perner et al., 2006). Due to the limited information we have on the localization of the optodes, however, we need to proceed cautiously when speculating about the involvement of specific brain areas and their lateralization in INS processes.

Interpersonal coordination on the neural level between mothers and their children was previously found to positively correlate with successful cooperation, namely higher task performance and communication (see Nguyen, Bánki, et al., 2020). INS during the present father–child cooperative task, however, did not seem to be related to task performance. This finding underscores that the neural dynamics in father–child problem solving can diverge from mother–child problem solving although the interaction would follow similar behavioral dynamics (Conner, Knight, & Cross, 1997).

Diverging from mother–child dyads (Nguyen et al., 2020a), we did not find an association between child agency and INS in father–child cooperative problem solving. The absence of this effect is somewhat unexpected since the role of child characteristics and behavior has been described as essential for INS in previous research (Nguyen et al., 2020a; Quiñones‐Camacho et al., 2019). Attempting to explain these diverging findings, we need to consider the age‐range in the so far available findings on interpersonal synchrony and its role as a mechanism of dynamic mutual adjustments of behavior and brain activity. In caregiver–infant interaction during the first year of life, behavioral and neural coordination are guided by non‐verbal communicative rhythms, such as touch, gaze, singing, and vocalizations, and these patterns were shown in both father– and mother–child pairs (Feldman, 2012). As language and symbolic thought emerge toward the second year of life, they become part of the repertoire of how fathers and mothers can establish interpersonal synchrony with their children (Keren, Feldman, Namdari‐Weinbaum, Spitzer, & Tyano, 2005). Children further mature in their ability to deliberately engage, both physiologically and socio‐emotionally, in social interactions with both parents during their preschool years (Harrist & Waugh, 2002). Concerning the role of child agency in parent–child interactions in that child age range, however, Hughes, Lindberg, and Devine (2018) showed that mothers’ support for children’s agency and autonomy may be higher than fathers’ support. The potential implication of this finding could be that child agency during father–child interactions in that child age range might not be a driving factor for fathers to engage in a reciprocal interaction with their child (Bureau et al., 2014). Within such father–child interactions, child agency would rather map children's engagement in the task itself, which may not be related to INS per se. In future studies, additional dyadic characteristics in association with parental gender should be taken into account to further explore and clarify potential similarities and differences in mother– versus father–child interaction, child agency, and INS.

Besides child agency, we also tested for associations between behavioral reciprocity and paternal sensitivity and INS. However, these two behavioral measures were not significantly related to INS, particularly during cooperation. In previous mother–child hyperscanning studies, interaction quality, in terms of synchrony/reciprocity, during problem solving was shown to positively correlate with INS in the TPJ and dlPFC (Nguyen et al., 2020a; Quiñones‐Camacho et al., 2019). Furthermore, Nguyen et al. (2020b) found increased INS to relate with higher amounts of turn‐taking during mother–child conversation. However, in the current study, father–child dyads showed rather low levels of reciprocity with limited variance between dyads. The lack of variance in reciprocity between fathers and their children may thus have resulted in a non‐significant relation of reciprocity with INS. The low levels of reciprocity further indicate that father–child joint problem solving was less likely to be coordinated in a turn‐taking manner. Even though this finding is in line with behavioral research that fathers are less likely to show cyclic interactions with their children (e.g., Lamb, 2010), it begs the question whether fathers establish INS with their children using different behavioral dynamics in comparison to mothers’ continuous adjustments throughout the interaction (see Markova et al., 2019). Future studies should look into the effect of experimentally manipulated levels of reciprocity in a task that necessitates turn‐taking behavior to examine how those affect INS.

Regarding parental sensitivity, our analyses did not reveal any association with INS during cooperation. This lack of association is consistent with previous work using the same problem‐solving task in mother–child pairs (Nguyen et al., 2020a). Although parental sensitivity did relate to another behavioral measure of interaction quality during both mother– and father–child interaction, reciprocity (see also below), such positive interactional parent characteristics did not seem to translate into INS. More research is needed to unveil whether this lack of association generalizes across other social contexts and age groups. For instance, parental sensitivity may play a more prominent role in more demanding or stressful contexts and/or for younger children.

It should also be mentioned here that no specific associations involving biological child sex during the cooperation condition were present. It was therefore not possible to extend previous observations of fathers supporting their sons’ agency by activating them (Paquette & Dumont, 2013) and fathers being more attuned toward their daughters and cater more toward their needs (e.g., de Mendonça et al., 2019) by means of INS in the current study. The issue of biological child sex differences, particularly during father–child interaction, nonetheless remains intriguing and should be further investigated using both behavioral as well as neuroimaging methods.

Apart from looking at associations between INS, behavioral task performance, and interaction quality from video ratings, we also assessed direct relations between the latter two behavioral measures. This revealed several interesting patterns. Behavioral task performance during cooperation was negatively related to paternal sensitivity but positively related to child agency and individual task performance. In addition, there was a positive association between paternal sensitivity and reciprocity.

Sensitive parenting in fathers was previously associated with more reciprocal behavior between fathers and their children (Harrist & Waugh, 2002; Leclère et al., 2014), and suggested to represent a marker for high interaction quality in terms of attachment security (Brown et al., 2012). In the present study, the pattern of results implies that sensitive parenting (with higher reciprocity) during cooperative problem solving was characterized by poorer task performance. In turn, higher cooperative task performance was linked to stronger child autonomy and individual task performance (i.e., child ability). Overall, these findings may suggest that father–child dyads were most successful in terms of cooperative task‐performance if fathers did not engage in sensitive and reciprocal behavior but instead the child was able to lead the task as the primary agent. Accordingly, task performance during cooperative interaction was determined by how well the children engaged in and subsequently solved the task by themselves, instead of depending on coordinating dyadic efforts.

Higher sensitivity in fathers was previously associated with engagement in didactic interactions (González, 1996), and INS evidenced during didactic interactions of children with their teacher was linked to learning success rather than cooperative problem‐solving success as an outcome (e.g., Bevilacqua et al., 2019). Consequently, cooperative task performance may only consider one style of interaction and thus capture only one out of many potential functions of (parent–child) INS (Hoehl et al., 2020). These considerations may potentially explain some of our findings reported here. While INS during father–child interaction was unrelated to cooperative task‐performance and child agency, cooperative task performance was related to both child agency and individual child ability. At the same time, this pattern does not imply a direct link between child ability (i.e., learning success) and parental sensitivity and/or reciprocity. It may be that in the present problem‐solving task, INS rather reflected the father’s observation of his child leading the task and only giving intermittent, not necessarily sensitive feedback—focusing on the child’s learning rather than the cooperation per se. The observed pattern differed from observations we made previously in mother–child pairs engaging in the same task where INS did relate to cooperative task performance and reciprocity (Nguyen et al., 2020a). In future studies, more than one task outcome to parent–child INS should be considered to take the caregiver’s interaction role, that is, as playmate, teacher, etc., into account. In addition, it would be very interesting to test children’s interactions with both their mothers and fathers, ideally in both dyadic and triadic settings. This would allow for investigating father–mother interaction patterns and relationship quality and their influence on parent–child interaction patterns and relationship quality (and vice‐versa).

Besides the influence of paternal sensitivity coded from interaction videos on INS, we observed that fathers’ self‐reported attitude toward their role as a parent (Role of the Father Questionnaire; ROFQ) was associated with the degree of INS in father–child problem solving. To date, this is the first fNIRS hyperscanning study to find such association. Father involvement is often mentioned as very important and conducive to fatherhood as well as child development (Flouri et al., 2016). Fathers’ attitude toward parenting has been shown to be a strong predictor for father involvement and more specifically parent–child interaction quality and quantity (Fox & Bruce, 2001). We assume that stronger identification with being a warm and supporting parent can help fathers in their self‐efficacy as well as sensitivity when interacting with their child (Brown et al., 2012). In particular, self‐efficacy is maintained to be an important mechanism to influence parenting behavior in terms of consistency, which feeds back to parents being able to engage in more harmonic interactions with their children (Giallo, Treyvaud, Cooklin, & Wade, 2013). Higher interaction quality through stronger identification with being a warm and supporting parent could thus be related to higher levels of INS in parent–child interactions (Nguyen et al., 2020a). Although our results are only preliminary to this vast field of parenting constructs, they underscore the potential factor of parental self‐efficacy for future studies concerning parent–child INS.

The current study has some limitations. One limitation is the homogeneity of the participants’ socio‐economic backgrounds. It may be that due to the lack of socio‐economic variance in our sample, our findings are more strongly representative of a certain caregiver group rather than easily generalizable to the overall population. For example, Allport et al. (2018) report that especially low‐resource families struggle with father involvement in child‐rearing, which calls for future studies addressing how interpersonal synchronization and parental characteristics interact in different, more varied socio‐economic groups. Another limitation is the relatively short interaction duration. The experimental conditions in the present study each lasted for 4 min in total, which is shorter than the intervals used in other studies that derive behavioral ratings from videos (e.g., Hirschmann et al., 2011). Accordingly, our coders had to evaluate the father–child interaction based on fewer observation points, which might have affected the rating results.

### Conclusions and Future Directions

This fNIRS hyperscanning study investigated INS during cooperative problem solving in father–child dyads as a function of dyadic behavioral and individual trait‐like variables in fathers for the first time. In line with existing results from dyadic fNIRS measurements in school‐aged child‐parent and preschool child‐mother dyads, we report increased INS during cooperation in lateral prefrontal (dlPFC) and temporoparietal (TPJ) brain areas in father–child dyads at preschool age. This said, our data tentatively suggest that INS during cooperative problem solving in father–child dyads may reflect somewhat different underlying processes as compared to those found in mother–child dyads. For example, our findings on INS in father–child dyads weakens the notion of child agency as a central behavioral variable relating to inter‐dyadic variability in INS, since it might play a more important role in mother–child than in father–child interaction. Moreover, we only observed an association between INS and cooperative task performance and reciprocity in mother– but not father–child pairs. Future studies might be able to decipher further relevant behaviors and traits in relation to INS in father–child interactions and probe possible mediation / moderation mechanisms (see Feng et al., 2020). A more detailed comparison of behavioral and INS patterns in dyads consisting of mothers and fathers with their children seems to be a promising research avenue. Specifically considering father–child interaction, our study identified fathers’ self‐reported attitude toward their role as a parent as a relevant factor for father–child INS during cooperation. In a broader societal context, it, therefore, seems relevant to promote the importance of paternal involvement by means of stronger identification with being a warm and supporting parent for child developmental outcomes.

## Supporting information

**Appendix S1.** Additional analyses and full model outputs
