# Supplementary material for: Interpersonal Neural Synchrony During Father–Child Problem Solving: An fNIRS Hyperscanning Study
Source: Child Dev. 2021 Jan 10;92(4):e565–80. doi: 10.1111/cdev.13510 (PMC8451924; doi:10.1111/cdev.13510)
Supplement: Supplementary file 1 — Appendix S1. Additional analyses and full model outputs [file CDEV-92-e565-s001.docx]

# **Appendix**

Linear Mixed Effect Model Formulae

*Model 1: wtc ~ condition + ROI + condition : ROI +*

*(1+condition | dyad)*

*Model 2: wtc ~ condition + cooperative task performance + behavioral reciprocity + ROI +*

*condition : cooperative task performance + condition : behavioral reciprocity +*

*(1 | dyad)*

*Model 3: wtc ~ condition + child agency + condition : child agency + ROI +*

*(1 | dyad)*

*Model 4: wtc ~ condition + paternal sensitivity + parental stress + ROI +*

*condition : paternal sensitivity + condition : parental stress +*

*(1 | dyad)*

*Model 5: wtc ~ condition + role of the father + ROI + condition : role of the father +*

*(1 | dyad)*

fNIRS Random Pair Analysis

To control for spurious correlations, we conducted an additional random pair analysis with 1,000 permutations. The coherence values of original dyads in each condition were tested against a distribution of randomized pair coherences in the same condition. Resulting p-values were corrected with a false discovery rate (FDR) for multiple comparisons (Benjamini & Hochberg, 1995). Results revealed that coherence values of original dyads for channels 1-8, 10 and 12-16 were significantly higher than the corresponding random pair distributions, *t*(999)=1.77-3.53, *q* < .05. Channels 9 and 11 did not show such significant differences in any conditions, *q* > .08. Yet, due to the clustering of channels in regions of interest we retained all channels for further statistical analyses.

Exploratory Analyses

In further analyses, we explored the role of biological child sex on the association between the assessed variables (i.e. cooperative task performance, behavioral reciprocity, child agency, paternal sensitivity, parental stress, and Role of the Father Questionnaire – ROFQ – scores) and INS.

*Biological Child Sex*

We re-analysed all models and included an interaction with biological child sex. Overall, the results showed that biological child sex was not related to INS during cooperation itself, *p* > .43, nor did it affect the association between assessed variables and INS, *p* > .087.

*Behavioral Analyses*

Descriptive variables for all behavioral ratings and self-reported variables are described in *Appendix Table A2*.

We tested associations between the dyad’s cooperative task performance and their behavior (i.e. sensitivity, agency, and individual task performance), biological child sex as well as self-report variables (ROFQ, and parental stress) during the problem-solving tasks in linear regression models. Results revealed the following significant associations:

- Cooperative task performance was significantly negatively related to paternal sensitivity during the interaction, β = -0.51, *p* <.001. Dyads with fathers who behaved responsively, warmly and encouragingly towards their child solved fewer tangram puzzles as compared to dyads with fathers who were less attuned to their child’s needs.
- Child agency was significantly positively correlated with cooperative task performance, β = 0.47, *p* < .01. The more the child was involved and leading the interaction, the more templates the dyad solved during the cooperation condition.
- Child individual task performance was positively associated with cooperative task performance, β = 0.39, *p* < .01, showing that the more puzzles the child solved in the individual condition, the more puzzles the dyad solved together.

In turn, reciprocity (*p* = .10) and the biological sex of the child (*p* = .44) were not significantly associated with cooperative task performance. Similarly, no significant effects emerged for the associations between cooperative task performance and ROFQ (*p* = .25), parental stress (*p* = .75), or paternal education (*p* = .56).

The relationships between reciprocity and the remaining behavioral variables (i.e. parental sensitivity, child agency, as well as self-reported ROFQ and stress) were examined with a zero-inflated Poisson regression model due to the left-skewed and zero-inflated distribution of reciprocity. Here, we found a significant positive relationship between reciprocity and parental sensitivity, β = 0.26, *p* < .03. More sensitive fathers showed more reciprocal behavior with their children. Also, fathers with higher education status seemed to show a marginal trend towards higher reciprocity displayed during the puzzle task, β = 0.13, *p* = .08. Conversely, analyses showed no associations between reciprocity and biological child sex (*p* = .47), as well as child agency (*p* = .77), ROFQ (*p* = .82), and parental stress (*p* = .41) during cooperative problem-solving.

Finally, we investigated the relationship between the father’s and child’s behavior as well as self-reported ROFQ using linear regression models. For a summary of results, please see *Appendix Table A3*.

fNIRS HbR Analysis

To test for whether INS differed between HbO and HbR, we also estimated Model 1 of the linear mixed effects models using INS values from HbR time-series. Firstly, we replicated the main effect of condition, χ²(2) = 16.40, *p* < .001, but only found marginal effect for region, χ²(3) = 7.80, *p* = .050, and the interaction effect between region and condition, χ²(6) = 11.31, *p* = .079. Post-hoc contrasts depicted significantly higher INS in the cooperation condition in comparison to the individual and rest conditions in the right dlPFC and left TPJ, *z* > 2.54, *p* < 0.029. For the left dlPFC and right TPJ, contrasts were not significant between conditions, *p* > .377.

Appendix Tables

Table A1. Model Outputs (Models 1 - 5)

| **Model 1: *wtc ~ condition + ROI + condition:ROI*** | | | | | | | |
| --- | --- | --- | --- | --- | --- | --- | --- |
| *Model Component* | *Estimates* | *SE* | *CI Lower* | *CI Upper* | χ*²* | *df* | *p* |
| (Intercept) | 0.351 | 0.005 | 0.343 | 0.360 |  |  |  |
| condition |  |  |  |  | 28.49 | 2 | <.001 |
| condition (individual) | -0.020 | 0.006 | -0.030 | -0.008 |  |  |  |
| condition (rest) | -0.023 | 0.006 | -0.035 | -0.010 |  |  |  |
| condition : ROI |  |  |  |  | 19.20 | 9 | .024 |
| condition (cooperation) : ROI (right dlPFC) | 0.006 | 0.005 | -0.003 | 0.016 |  |  |  |
| condition (cooperation) : ROI (left TPJ) | 0.007 | 0.005 | -0.001 | 0.016 |  |  |  |
| condition (cooperation) : ROI (right TPJ) | -0.006 | 0.004 | -0.015 | 0.003 |  |  |  |
| condition (individual) : ROI (right dlPFC) | -0.002 | 0.005 | -0.011 | 0.007 |  |  |  |
| condition (individual) : ROI (left TPJ) | 0.002 | 0.005 | -0.006 | 0.011 |  |  |  |
| condition (individual) : ROI (right TPJ) | 0.002 | 0.004 | -0.006 | 0.011 |  |  |  |
| condition (rest) : ROI (right dlPFC) | 0.001 | 0.005 | -0.008 | 0.010 |  |  |  |
| condition (rest) : ROI (left TPJ) | 0.009 | 0.005 | 0.001 | 0.020 |  |  |  |
|  | *Estimates* | *SE* | *CI Lower* | *CI Upper* | χ*²* | *df* | *p* |
| condition (rest) : ROI (right TPJ) | 0.002 | 0.004 | -0.006 | 0.012 |  |  |  |
| **Model 2: *wtc ~ condition+ cooperative task performance + behavioral reciprocity + ROI +***  ***condition : cooperative task performance + condition : behavioral reciprocity*** | | | | | | | |
| *Model Component* | *Estimates* | *SE* | *CI Lower* | *CI Upper* | χ*²* | *df* | *p* |
| (Intercept) | 0.351 | 0.005 | 0.341 | 0.361 |  |  |  |
| condition |  |  |  |  | 68.89 | 1 | <.001 |
| condition (individual) | -0.020 | 0.002 | -0.026 | -0.016 |  |  |  |
| task performance | 0.004 | 0.002 | -0.001 | 0.009 | 0.63 | 1 | .426 |
| behavioral reciprocity | 0.001 | 0.003 | -0.003 | 0.007 | 0.45 | 1 | .498 |
| ROI |  |  |  |  | 4.25 | 3 | .235 |
| ROI (left TPJ) | 0.004 | 0.004 | -0.002 | 0.011 |  |  |  |
| ROI (right dlPFC) | 0.002 | 0.004 | -0.004 | 0.009 |  |  |  |
| ROI (right TPJ) | -0.002 | 0.003 | -0.008 | 0.005 |  |  |  |
| condition : task performance |  |  |  |  | 2.37 | 1 | .123 |
| condition (individual) : task performance | 0.000 | 0.002 | -0.004 | 0.005 |  |  |  |
| condition : behavioral reciprocity |  |  |  |  | 0.08 | 1 | .771 |
| condition (individual) : behavioral reciprocity | 0.000 | 0.002 | -0.004 | 0.005 |  |  |  |
| **Model 3: *wtc ~ condition + child agency + ROI + condition : child agency*** | | | | | | | |
| *Model Component* | *Estimates* | *SE* | *CI Lower* | *CI Upper* | χ*²* | *df* | *p* |
| (Intercept) | 0.351 | 0.003 | 0.345 | 0.358 |  |  |  |
| condition |  |  |  |  | 68.84 | 1 | <.001 |
| condition (individual) | -0.020 | 0.002 | -0.026 | -0.016 |  |  |  |
| child agency | 0.004 | 0.003 | -0.001 | 0.009 | 1.28 | 1 | .257 |
| ROI |  |  |  |  | 4.29 | 3 | .231 |
| ROI (left TPJ) | 0.004 | 0.004 | -0.002 | 0.011 |  |  |  |
| ROI (right dlPFC) | 0.002 | 0.004 | -0.004 | 0.009 |  |  |  |
| ROI (right TPJ) | -0.002 | 0.003 | -0.008 | 0.005 |  |  |  |
| condition : child agency |  |  |  |  | 1.50 | 2 | .221 |
| condition (individual) : child agency | -0.003 | 0.002 | -0.008 | 0.002 |  |  |  |
| **Model 4: *wtc ~ condition + paternal sensitivity + parental stress + ROI +***  ***condition : paternal sensitivity + condition : parental stress*** | | | | | | | |
| *Model Component* | *Estimates* | *SE* | *CI Lower* | *CI Upper* | χ*²* | *df* | *p* |
| (Intercept) | 0.352 | 0.003 | 0.347 | 0.357 |  |  |  |
| condition |  |  |  |  | 68.89 | 1 | <.001 |
| condition (individual) | -0.021 | 0.003 | -0.027 | -0.016 |  |  |  |
| paternal sensitivity | 0.002 | 0.002 | -0.003 | 0.007 | 0.33 | 1 | .572 |
| parental stress | -0.004 | 0.003 | -0.009 | 0.001 | 2.82 | 1 | .092 |
| ROI |  |  |  |  | 3.06 | 3 | .381 |
| ROI (left TPJ) | 0.004 | 0.004 | -0.002 | 0.011 |  |  |  |
| ROI (right dlPFC) | 0.002 | 0.004 | -0.004 | 0.009 |  |  |  |
| ROI (right TPJ) | -0.002 | 0.003 | -0.008 | 0.005 |  |  |  |
| condition : paternal sensitivity |  |  |  |  | 0.28 | 1 | .596 |
| condition (individual) : paternal sensitivity | 0.001 | 0.003 | -0.007 | 0.004 |  |  |  |
| condition : parental stress |  |  |  |  | 0.00 | 1 | .996 |
| condition (individual) : parental stress | 0.001 | 0.003 | -0.005 | 0.005 |  |  |  |
| **Model 5: *wtc ~ condition + role of the father + ROI + condition : role of the father*** | | | | | | | |
| *Model Component* | *Estimates* | *SE* | *CI Lower* | *CI Upper* | χ*²* | *df* | *p* |
| (Intercept) | 0.352 | 0.003 | 0.345 | 0.358 |  |  |  |
| condition |  |  |  |  | 68.84 | 1 | <.001 |
| condition (individual) | -0.021 | 0.002 | -0.026 | -0.016 |  |  |  |
| role of the father | 0.007 | 0.002 | 0.002 | 0.012 | 4.58 | 1 | .032 |
| ROI |  |  |  |  | 3.17 | 3 | .365 |
| ROI (left TPJ) | 0.004 | 0.004 | -0.003 | 0.012 |  |  |  |
| ROI (right dlPFC) | 0.003 | 0.004 | -0.003 | 0.010 |  |  |  |
| ROI (right TPJ) | -0.001 | 0.003 | -0.009 | 0.006 |  |  |  |
| condition : role of the father |  |  |  |  | 3.59 | 1 | .058 |
| condition (individual) : role of the father | -0.005 | 0.002 | -0.010 | 0.000 |  |  |  |

*Note.*

(1) The factor condition was dummy coded and had the cooperation condition as reference level Estimates for the single predictors indicate the change from the response when the predictor changes from the reference level to the level of the predictor (in parentheses). Only estimates concerning the cooperation condition are reported.

(2) Confidence intervals were derived using parametric bootstraps using the function confint.merMod

Included are Estimates, standard errors (SE), confidence intervals (CI), and likelihood ratio test outputs for the single effects.

| Table A2. Descriptives for Behavioral Analyses | | | | | | | | | | | | | | | |
| --- | --- | --- | --- | --- | --- | --- | --- | --- | --- | --- | --- | --- | --- | --- | --- |
|  | | **BR** | | **PS** | | **CA** | | **Coop. TP** | | **Indiv. TP** | | **ROFQ** | | **EBI** | |
| N |  | 68 |  | 68 |  | 68 |  | 68 |  | 68 |  | 60 |  | 61 |  |
| Mean |  | 1.89 |  | 3.54 |  | 3.85 |  | 2.76 |  | 0.80 |  | 4.08 |  | 110 |  |
| SD |  | 0.90 |  | 1.09 |  | 1.40 |  | 1.38 |  | 1.05 |  | 0.44 |  | 23.8 |  |
| Min |  | 1.00 |  | 1.00 |  | 1.00 |  | 0 |  | 0 |  | 2.67 |  | 65 |  |
| Max |  | 5.00 |  | 6.00 |  | 7.00 |  | 6 |  | 5 |  | 4.80 |  | 170 |  |
| *Note. BR=Behavioral Reciprocity, PS=Paternal sensitivity, CA=Child agency, Coop.=Cooperation, Indiv.=Individual; TP=Task performance, ROFQ=Role of the Father Questionnaire, EBI=Parental Stress Index* | | | | | | | | | | | | | | | |

| Table A3. Correlation Matrix for Behavioral Analyses | | | | | | | | | | | |
| --- | --- | --- | --- | --- | --- | --- | --- | --- | --- | --- | --- |
|  | |  | | **1** | | **2** | | **3** | | **4** | |
| Paternal sensitivity |  | Pearson's r |  | — |  |  |  |  |  |  |  |
|  |  | p-value |  | — |  |  |  |  |  |  |  |
| Child agency |  | Pearson's r |  | 0.606 | *** | — |  |  |  |  |  |
|  |  | p-value |  | < .001 |  | — |  |  |  |  |  |
| EBI |  | Pearson's r |  | -0.075 |  | -0.227 |  | — |  |  |  |
|  |  | p-value |  | 0.567 |  | 0.079 |  | — |  |  |  |
| ROFQ |  | Pearson's r |  | 0.290 | * | 0.192 |  | -0.469 | *** | — |  |
|  |  | p-value |  | 0.024 |  | 0.141 |  | < .001 |  | — |  |
| *Note.* Parental Stress Index (Eltern-Belastungs-Inventar; EBI), Role of the Father Questionnaire (ROFQ). * p < .05, *** p < .001. There was a significant positive relationship between paternal sensitivity and child agency as well as ROFQ, and a significant negative relationship between parental stress and ROFQ. | | | | | | | | | | | |
|  | | | | | | | | | | | |


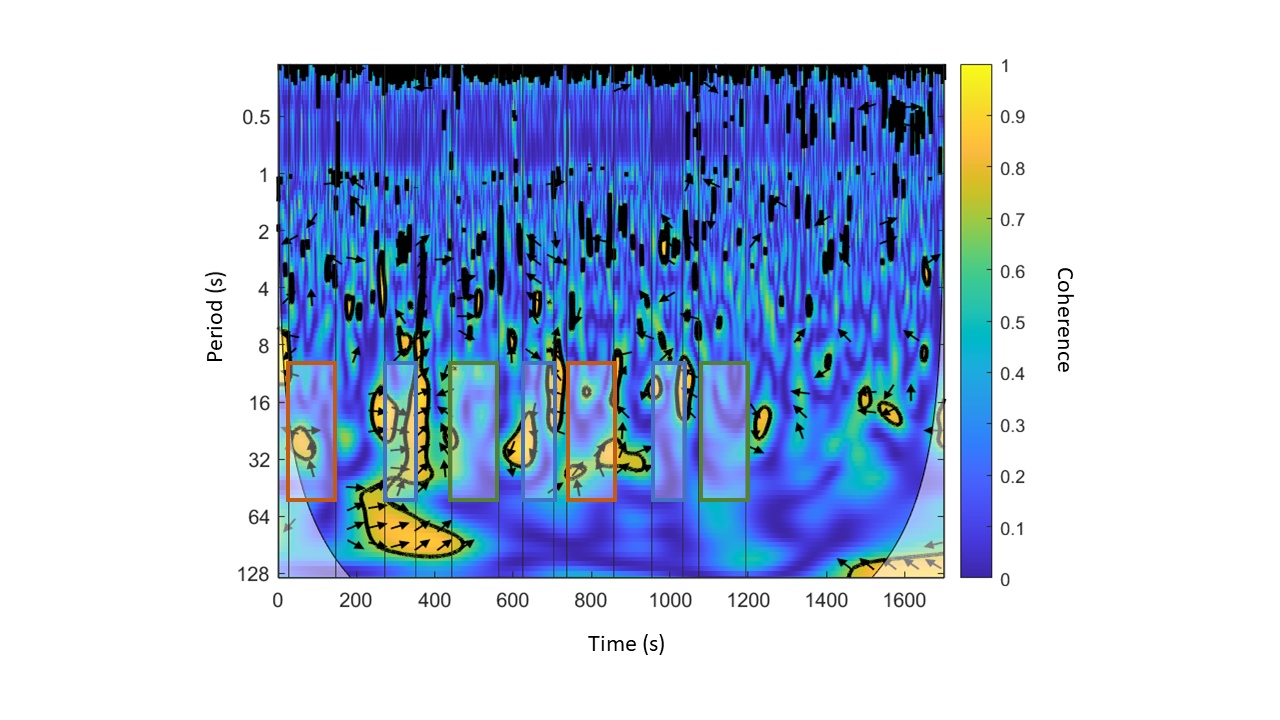


*Figure A1*. The illustration depicts an exemplary wavelet transform coherence (WTC) plot between two homologous channels in a father-child dyad. Coherence is calculated over time (x-axis) and over different frequencies, i.e. periods (y-axis). Coherence values are indicated in the color bar on the right-hand side. For further statistical analyses, coherence values are averaged over the frequency band of interest (10-50 period seconds) and the length of the condition (red square=cooperation, green square=individual, blue square=rest) for each channel pair in each dyad.

Bibliography

Benjamini, Y., & Hochberg, Y. (1995). Controlling the False Discovery Rate: A Practical and Powerful Approach to Multiple Testing. *Journal of the Royal Statistical Society: Series B (Methodological)*, *57*(1), 289–300. doi: [10.1111/j.2517-6161.1995.tb02031.x](https://doi.org/10.1111/j.2517-6161.1995.tb02031.x)
